# An Intra-Individual Comparison of MRI, [^18^F]-FET and [^18^F]-FLT PET in Patients with High-Grade Gliomas

**DOI:** 10.1371/journal.pone.0095830

**Published:** 2014-04-23

**Authors:** Martha Nowosielski, Matthew D. DiFranco, Daniel Putzer, Marcel Seiz, Wolfgang Recheis, Andreas H. Jacobs, Günther Stockhammer, Markus Hutterer

**Affiliations:** 1 Department of Neurology, Innsbruck Medical University, Innsbruck, Austria; 2 Department of Oto-, Rhino- and Laryngology with 4D Visualization Lab, Innsbruck Medical University, Innsbruck, Austria; 3 Department of Nuclear Medicine, Innsbruck Medical University, Innsbruck, Austria; 4 Department of Neurosurgery, Innsbruck Medical University, Innsbruck, Austria; 5 Department of Radiology, Innsbruck Medical University, Innsbruck, Austria; 6 Department of Radiology, Computational Image Analysis and Radiology Lab (CIR), Medical University of Vienna, Vienna, Austria; 7 Department of Neurology and Wilhelm-Sander Neurooncology Unity, University Hospital and Medical School Regensburg, Regensburg, Germany; 8 European Institute for Molecular Imaging (EIMI) at the Westphalian Wilhelms University, Münster, Germany; 9 Department of Geriatrics at Evangelische Kliniken, Johanniter Krankenhaus, Bonn, Germany; 10 Department of Neurosurgery, Mannheim Medical University, Mannheim, Germany; City of Hope, United States of America

## Abstract

**Objectives:**

Intra-individual spatial overlap analysis of tumor volumes assessed by MRI, the amino acid PET tracer [^18^F]-FET and the nucleoside PET tracer [^18^F]-FLT in high-grade gliomas (HGG).

**Methods:**

MRI, [^18^F]-FET and [^18^F]-FLT PET data sets were retrospectively analyzed in 23 HGG patients. Morphologic tumor volumes on MRI (post-contrast T1 (cT1) and T2 images) were calculated using a semi-automatic image segmentation method. Metabolic tumor volumes for [^18^F]-FET and [^18^F]-FLT PETs were determined by image segmentation using a threshold-based volume of interest analysis. After co-registration with MRI the morphologic and metabolic tumor volumes were compared on an intra-individual basis in order to estimate spatial overlaps using the Spearman's rank correlation coefficient and the Mann-Whitney U test.

**Results:**

[^18^F]-FLT uptake was negative in tumors with no or only moderate contrast enhancement on MRI, detecting only 21 of 23 (91%) HGG. In addition, [^18^F]-FLT uptake was mainly restricted to cT1 tumor areas on MRI and [^18^F]-FLT volumes strongly correlated with cT1 volumes (r = 0.841, p<0.001). In contrast, [^18^F]-FET PET detected 22 of 23 (96%) HGG. [^18^F]-FET uptake beyond areas of cT1 was found in 61% of cases and [^18^F]-FET volumes showed only a moderate correlation with cT1 volumes (r = 0.573, p<0.001). Metabolic tumor volumes beyond cT1 tumor areas were significantly larger for [^18^F]-FET compared to [^18^F]-FLT tracer uptake (8.3 vs. 2.7 cm^3^, p<0.001).

**Conclusion:**

In HGG [^18^F]-FET but not [^18^F]-FLT PET was able to detect metabolic active tumor tissue beyond contrast enhancing tumor on MRI. In contrast to [^18^F]-FET, blood-brain barrier breakdown seems to be a prerequisite for [^18^F]-FLT tracer uptake.

## Introduction

Magnetic resonance imaging (MRI), as the gold standard diagnostic tool for brain tumors, offers high spatial resolution and is widely available [1]. In high-grade gliomas (HGG) the area of contrast enhancement on MRI T1-weighted sequences is generally assumed to reflect the main tumor burden [1]. In neuropathologic studies, however, invasive glioma cells can be found far beyond contrast enhancing areas [2]–[5]. Recently, molecular imaging studies using the amino acid tracers [^11^C]-MET and [^18^F]-FET revealed that in HGG patients the “metabolic tumor volumes” are frequently larger on PET compared to the corresponding “morphologic contrast enhancing tumor volumes” on MRI. This observation indicates that the main tumor burden may be substantially underestimated on standard MRI [6], [7].

O-(2-[^18^F]-fluoro-Ethyl)-L-tyrosine ([^18^F]-FET) is an amino acid tracer frequently used in the management of glial brain tumors [8]. [^18^F]-FET uptake correlates with tumor cell density and proliferation rate as well as with microvascular density. From clinical studies there is increasing evidence for the practical value of [^18^F]-FET PET in addition to MRI. Complete resection guided by [^18^F]-FET tracer uptake in HGG increased overall survival [9] and [^18^F]-FET PET-based radiotherapy planning in HGG improved target volume definition [10], [11] and improved surgery planning in low-grade gliomas for hot spot detection with static images [12], [13] and dynamic acquisition methods [14]. For treatment monitoring [^18^F]-FET PET enabled earlier detection of tumor progression after concomitant chemo-/radiotherapy [15], during adjuvant chemotherapy [16], [17], and in the course of anti-angiogenic therapy [18] and local treatment strategies [19], [20].

A second [^18^F]-labeled PET tracer also increasingly used in brain tumors is [^18^F]-3′-fluoro-3′-deoxy-L-thymidine ([^18^F]-FLT), a radiolabeled fluorinated thymidine analog, which shows good correlation with the Ki-67 proliferation rate in patients with newly diagnosed HGG [21], [22].

Currently, little is known about how [^18^F]-FET and [^18^F]-FLT directly compare to each other and to MRI in individual HGG patients. Therefore, the objective of this retrospective study was an intra-individual comparison of MRI cT1 and T2 sequences to the corresponding [^18^F]-FET and [^18^F]-FLT tracer uptakes in patients with HGG to better determine the spatial overlap and the practical value of these nuclear imaging modalities. We quantitatively assessed lesion-to-brain uptake ratios in PET as well as PET-based metabolic and MR-based morphologic tumor volumes and calculated territorial overlaps using a three-dimensional volumetric approach.

## Material and Methods

### Patient population

Patients gave written informed consent to both PET and MRI investigations during routine diagnostic procedure. The ethics committee of Innsbruck Medical University approved the retrospective data evaluation of imaging and clinical data from those patients. All data were stored in the clinics' database. The ethics committee waived the need for another written informed consent from those patients to retrospectively analyze their data.

In this study 23 patients (15 men and 8 women; mean age 54 years, range 36–81 years) with histologically confirmed HGG, who underwent timely corresponding MRI, [^18^F]-FET and [^18^F]-FLT PET examinations at primary diagnosis (n = 3) or tumor progression (n = 20), were included. The mean time intervals between the imaging studies were 5.9 days for [^18^F]-FET/[^18^F]-FLT PET, 13.1 days for MRI/[^18^F]-FET PET and 11.0 days for MRI/[^18^F]-FLT PET.

Histological diagnoses of the study population revealed glioblastoma multiforme WHO IV (GBM; n = 16), anaplastic astrocytoma WHO III (AA, n = 5), anaplastic oligodendroglioma WHO III (AO, n = 2). All three newly diagnosed patients had no treatment prior to imaging. In 20 HGG patients who were diagnosed with tumor recurrence (14 patients with first tumor recurrence, 5 with a second tumor recurrence and 1 patient with third tumor recurrence), treatment prior to imaging included surgery in all patients (19 macroscopic total resections, 1 subtotal tumor resections and 3 stereotactic biopsies), radiation and temozolomide chemotherapy according to the EORTC 26981/22981 NCIC CE.3 protocol [23] in 19 patients; one patient was treated with adjuvant PCV chemotherapy. Mean time interval from radiation until tumor progression was 1.9 years (range 0.1–8.3 years). At the time of neuroimaging 6 patients received corticosteroids (dexamethasone dose ranging between 2 mg and 12 mg daily). None of the patients had previous treatment with bevacizumab. Individual clinical data are summarized in Table 1.

**Table 1:** Patient population.

Pat	Sex^a^	Age^b^	TU-Type WHO grade^c^	Location^d^	Treatment prior to study analysis			Histology	CS^h^ [mg]
					Surgery^e^	Radiation^f^	Chemotherapy^g^		
1	M	54	GBM IV	LR-occipital	MTR	60Gy	TMZ	+	2
2	F	58	GBM IV	L-temporal	MTR x 2	60Gy	TMZ	+	12
3	M	57	GBM IV	L-temporal	MTR	60Gy	TMZ	+	12
4	F	40	GBM IV	R-temporal	MTR	60Gy	TMZ, SUT, TMZ	+	-
5	F	38	GBM IV	L-occipital	MTR	60Gy	TMZ	+	12
6	M	49	GBM IV	L-temporal	MTR	-	-	+	-
7	M	54	GBM IV	LR-frontal	STR	-	-	+	-
8	M	64	GBM IV	L-frontal	MTR x2	60Gy	TMZ	+	-
9	M	45	GBM IV	L-frontal	MTR	60Gy	TMZ	+	2
10	M	75	GBM IV	R-parietal	MTR	60Gy	TMZ	+	-
11	F	53	GBM IV	R-parietal	MTR x 2	60Gy	TMZ	+	-
12	M	50	GBM IV	R-parietal	MTR	60Gy	TMZ	+	4
13	F	64	GBM IV	R-parietal	MTR	60Gy	TMZ	+	-
14	F	78	GBM IV	R-parietal	MTR	-	-	+	-
15	M	36	GBM IV	R-frontal	MTR	60Gy	TMZ	+	-
16	M	45	GBM IV	L-temporal	MTR	60Gy, 54Gy	TMZ	+	-
17	M	41	AA III	R-temporal	MTR	-	-	+	-
18	M	62	AA III	R-frontal	MTR	60Gy	TMZ	+	-
19	F	43	AA III	R-frontal	MTR	60Gy	TMZ	+	-
20	M	81	AA III	R-temporal	STB	60Gy	TMZ	+	-
21	M	61	AA III	L-parietal	STB	60Gy	TMZ	+	-
22	M	54	AO III	L-parietal	STB	60Gy	TMZ	+	-
23	M	47	AO III	R-parietal	MTR	60Gy	PCV, TMZ	+	-

a M = male, F = female.

b age at first diagnosis.

c tumor grade according WHO classification; GBM = glioblastoma multiforme, AA = anaplastic astrocytoma, AO = anaplastic oligodendroglioma.

d L = left hemisphere, R = right hemisphere, LR = both hemispheres affected.

e MTR = macroscopic total resection, STR = subtotal resection, STB = stereotactic biopsy.

f Radiation; extended tumor field according to reference [23], Gy = Gray.

g TMZ = Temozolomide, SUT = Sunitinib malate, PCV = Procarbazine, Lomustine (CCNU), Vincristine.

h CS = Corticosteroids at time of imaging.

### MR imaging protocol

MRI studies were conducted on a 1.5 Tesla scanner (Sonata, Siemens-Erlangen, Germany) and included T1-weighted (TR = 1860 ms, TE = 4.38 ms with 1.2 mm slice thickness, 256×192 matrix), T2-weighted and fast-spin echo (6600 ms/100-110 ms, 2 mm slice thickness, 320×240 matrix) sequences. Post-contrast T1-weighted images were acquired 5 minutes after contrast agent injection (Omniscan, Dotarem, 0.1 mmol/kg).

### PET imaging protocol

[^18^F]-FET and [^18^F]-FLT PET scans were conducted on a PET/CT scanner (GE Discovery PET/CT 690) using a transaxial reconstruction matrix of 256 × 256 (1 mm per pixel) and 47 axial slices with 3.27 mm. A low dose CT was performed as transmission scan. The mean standard dose for [^18^F]-FET of the brain was 238 MBq (range 180–320 MBq), while for [^18^F]-FLT it was 180 MBq (range 138–230 MBq). Radiation dosimetries for [^18^F]-FET and [^18^F]-FLT were described previously by Pauleit et al. [12], [24] and Vesselle et al. [25].

The radio-labeling yield and the radiochemical purity (95% level) had been previously controlled by the manufacturing company. Application of the [^18^F]-FET and [^18^F]-FLT tracers was done intravenously. [^18^F]-FET emission scans commenced mean 33.2 (range 23.3 to 47.6) minutes after tracer application and patient positioning within the gantry. Mean [^18^F]-FLT time interval was 37 minutes (range 24.3 to 55) minutes, respectively. The scan duration was five minutes. Images were acquired in a three-dimensional mode in contiguous transaxial slices of the entire brain. An iterative reconstruction of the attenuation-corrected emission data set was obtained using the ordered subset expectation maximization (OSEM) algorithm.

### Image registration

Registration of MRI (cT1 and T2), [^18^F]-FET and [^18^F]-FLT PET data sets was performed using the fast rigid registration package in Slicer 3D Version 3.6.3.1.0 [26]. All data were co-registered to MRI cT1 imaging sequence.

### MRI segmentation

MRI segmentation and morphologic tumor volume calculation of cT1 and T2 images were performed using a semi-automated active contour method (snake evolution, ITK-SNAP 2.0). This software has already demonstrated excellent reliability and efficacy of 3D segmentation [27].

### PET image analysis and segmentation

To estimate [^18^F]-FET and [^18^F]-FLT tracer uptakes and metabolic tumor volumes, the established semi-quantitative standard uptake value (SUV) calculation analysis [28] was performed by an in-house software package developed in Matlab [29]. To measure the maximal tracer uptake activity (SUVmax) spherical region of interest (ROI) volumes covering the maximal extension of the tumor were drawn in the affected brain hemisphere. To determine the background activity (SUV background) a spherical ROI volume was placed on the contralateral hemisphere (mirror region) including white and gray matter but not the ventricle (ROI volume ranged between 1.7 cm^3^ to 2 cm^3^). Afterwards, the lesion-to-background ratios (LBRs) were calculated by dividing SUVmax/SUV background and used for further volumetric and statistical analysis. For determination of the metabolic tumor volumes, LBR cut-off values were calculated using the receiver operating characteristic (ROC) curve analysis [30]. For [^18^F]-FET PET an LBR >1.62 (AUC 1.0; sensitivity 100% and specificity 100%) and for [^18^F]-FLT PET an LBR >1.69 (AUC 0.958±0.041; sensitivity 96% and specificity 100%) served as the optimal thresholds. LBRs above these cut-off levels were considered to be metabolically active tumor and were used for image segmentation and tumor volume calculation.

Afterwards a comparison of the spatial relationship between the metabolic PET and morphologic MRI volumes was performed using an overlap statistic calculation for two volumes. Importantly, due to the possible influence of a partial-volume effect on small residual tumor volumes on [^18^F]-FET and [^18^F]-FLT PET, metabolic tumor volumes below 2 cm^3^ were considered as not significant and were not taken into account for further analysis [31].

### Statistics

Data were analyzed using SPSS 20.0 statistical software. Testing on normal distribution was performed with the Kolmogorov-Smirnov-Test. Data not distributed normally were further analyzed by the Spearman correlation coefficient in order to detect correlations between tumor volumes and LBRs. The Mann-Whitney U test (MWU) was performed to detect differences in tumor volumes and WHO grading. Data are expressed in mean ± (standard error). Probability values <0.05 were considered as significant.

## Results

### Tumor detection with [^18^F]-FET and [^18^F]-FLT PET

**Figure 1:**
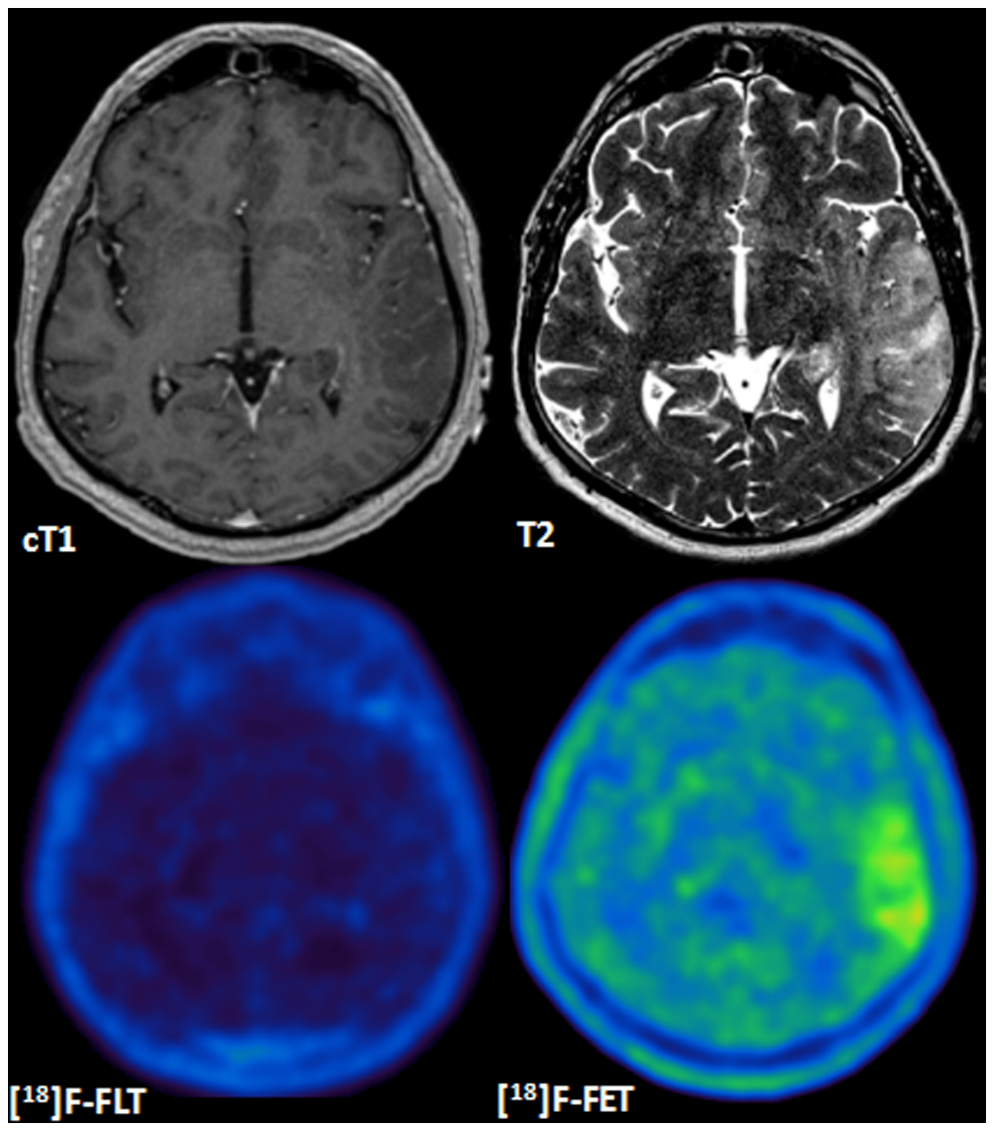
MRI and PET in a patient with non-enhancing GBM WHO IV. Newly diagnosed GBM WHO IV presenting with a hyperintense T2 lesion left parieto-temporal but without contrast enhancement on cT1. In contrast to the absence of [^18^F]-FLT tracer uptake, a metabolically active lesion can clearly be depicted on [^18^F]-FET PET.

The sensitivity for the detection of a HGG was higher for [^18^F]-FET compared to [^18^F]-FLT PET. [^18^F]-FLT was able to detect 21 of 23 HGG (91%) and tracer uptake was negative in tumors with no or moderate contrast enhancement (n = 2), including a GBM IV and an AA III (Figure 1). In contrast, [^18^F]-FET uptake was found in 22 of 23 HGG (96%). The [^18^F]-FET negative patient had an AA III with no contrast enhancement on MRI and was also negative on [^18^F]-FLT PET.

### [^18^F]-FET and [^18^F]-FLT LBRs and tumor volume calculations

**Figure 2:**
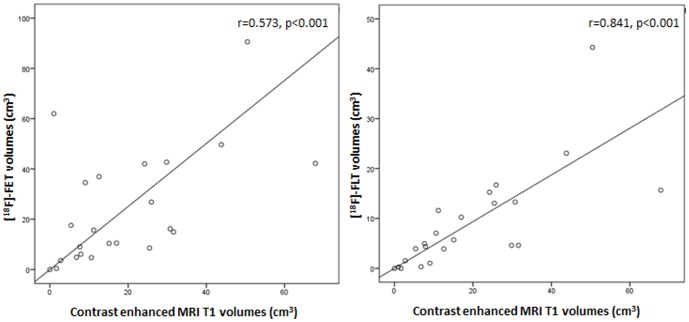
Correlation of PET with cT1 tumor volumes. The mean tumor volumes (cm^3^) were 23.9±4.8 for [^18^F]-FET PET, 8.9±2.0 for [^18^F]-FLT and 20.56±3.94 on contrast enhanced T1 sequences (cT1). [^18^F]-FLT volumes strongly correlated with cT1 (r = 0.841, p<0.001, respectively). [^18^F]-FET volumes showed a moderate correlation with cT1 (r = 0.573, p<0.001), Spearman correlation.

Mean LBR +/− standard error for [^18^F]-FET was 2.38±0.2 compared to [^18^F]-FLT with 3.08±0.18. No correlation was found between the uptake ratio of [^18^F]-FET and [^18^F]-FLT (r = 0.376, p = 0.07). Mean morphologic and metabolic tumor volumes were 20.6±3.9 cm^3^ on cT1, 146.6±17.6 cm^3^ on T2, 23.9±4.8 cm^3^ on [^18^F]-FET and 8.9±2.1 cm^3^ on [^18^F]-FLT PET, respectively. [^18^F]-FLT volumes correlated strongly with cT1 volumes (r = 0.841, p<0.001) and moderately with [^18^F]-FET volumes (r = 0.474, p = 0.02). In contrast, [^18^F]-FET volumes showed only a moderate correlation with cT1 volumes (r = 0.573, p<0.001, Figure 2). [^18^F]-FLT and [^18^F]-FET showed no correlation with T2 volumes (r = 0.358, p = 0.09 and r = 0.276, p = 0.18, respectively). Individual tumor volumes are listed in Table 2.

**Table 2:** Individual tumor volumes and tracer uptake.

Pat	Volumes				[^18^F]-FET outside			[^18^F]-FLT outside			Tracer uptake	
	(cm^3^)^a^				(cm^3^)^b^			(cm^3^)^c^			LBR^d^	
	cT1	T2	[^18^F]-FET	[^18^F]-FLT	cT1	T2	[^18^F]-FLT	cT1	T2	[^18^F]-FET	[^18^F]-FET	[^18^F]-FLT
1	10.6	151.6	4.7	7.0	0.1	0.0	0.3	0.2	0.0	2.6	1.63	2.89
2	31.6	155.0	14.9	4.6	16.9	5.0	33.5	0.0	0.0	0.0	2.59	2.39
3	50.5	223.1	90.6	44.3	24.0	2.6	46.5	2.7	0.65	0.3	3.90	5.40
4	9.0	30.3	34.6	1.0	16.9	5.0	33.5	0.0	0.0	0.0	2.59	2.39
5	7.9	36.4	6.1	4.4	3.6	2.9	4.6	0.3	1.7	2.9	1.73	3.65
6	1.7	63.4	0.4	0.0	0.4	0.0	0.4	0.0	0.0	0.0	1.64	1.13
7	67.9	106.2	42.2	15.7	9.8	7.0	28.8	0.5	1.1	2.2	2.68	2.77
8	30.7	108.8	16.2	13.3	1.5	0.2	3.5	0.3	0.1	0.6	2.56	3.83
9	2.8	39.8	3.6	1.5	1.4	0.1	2.5	0.2	0.4	0.4	1.93	3.84
10	11.2	74.4	15.7	11.6	5.0	1.9	7.0	1.1	0.4	2.9	1.96	3.16
11	24.2	147.4	42.1	15.2	8.9	2.8	26.8	0.0	0.0	0.0	2.59	2.81
12	25.5	265.0	8.5	13.0	0.3	0.0	0.5	0.1	0.0	5.0	2.02	4.07
13	43.8	335.8	49.7	23.1	9.6	2.9	26.8	1.5	0.8	0.1	2.73	3.32
14	5.4	108.2	17.6	3.9	9.2	0.3	13.7	0.1	0.0	0.0	2.05	2.93
15	25.9	95.6	26.8	16.7	1.8	0.4	10.5	0.3	0.2	0.4	2.70	3.25
16	6.8	227.4	4.8	0.3	1.6	0.0	4.7	0.0	0.0	0.2	1.69	2.19
17	0.0	72.6	0.0	0.0	0.0	0.0	0.0	0.0	0.0	0.0	1.13	1.23
18	7.7	179.2	9.0	4.9	2.8	0.0	5.6	0.0	0.1	1.5	1.97	3.21
19	15.1	131.1	10.4	5.7	3.2	0.2	7.8	0.0	0.0	3.2	1.83	3.08
20	12.6	263.9	36.9	3.9	17.1	2.6	33.0	0.7	0.1	0.0	5.86	5.18
21	29.8	142.5	42.7	4.6	14.0	0.7	38.3	0.2	0.1	0.2	2.79	2.74
22	1.0	125.3	62.0	0.3	59.2	0.2	61.7	0.2	0.0	0.0	3.21	2.07
23	17.0	122.8	10.5	10.2	0.5	0.1	1.0	0.4	0.1	0.7	2.23	5.04

a cT1 =  contrast enhanced T1 MRI volume.

b [^18^F]-FET =  [^18^F]-FET volume according a LBR threshold of > 1.62, detected beyond the margins of cT1, T2 and [^18^F]-FLT.

c [^18^F]-FLT =  [^18^F]-FLT volume according a LBR threshold of > 1.69, detected beyond the margins of cT1, T2 and [^18^F]-FET.

d LBR =  lesion to background ratio.

### Tracer uptake beyond the borders of cT1 and T2 on MRI

**Figure 3:**
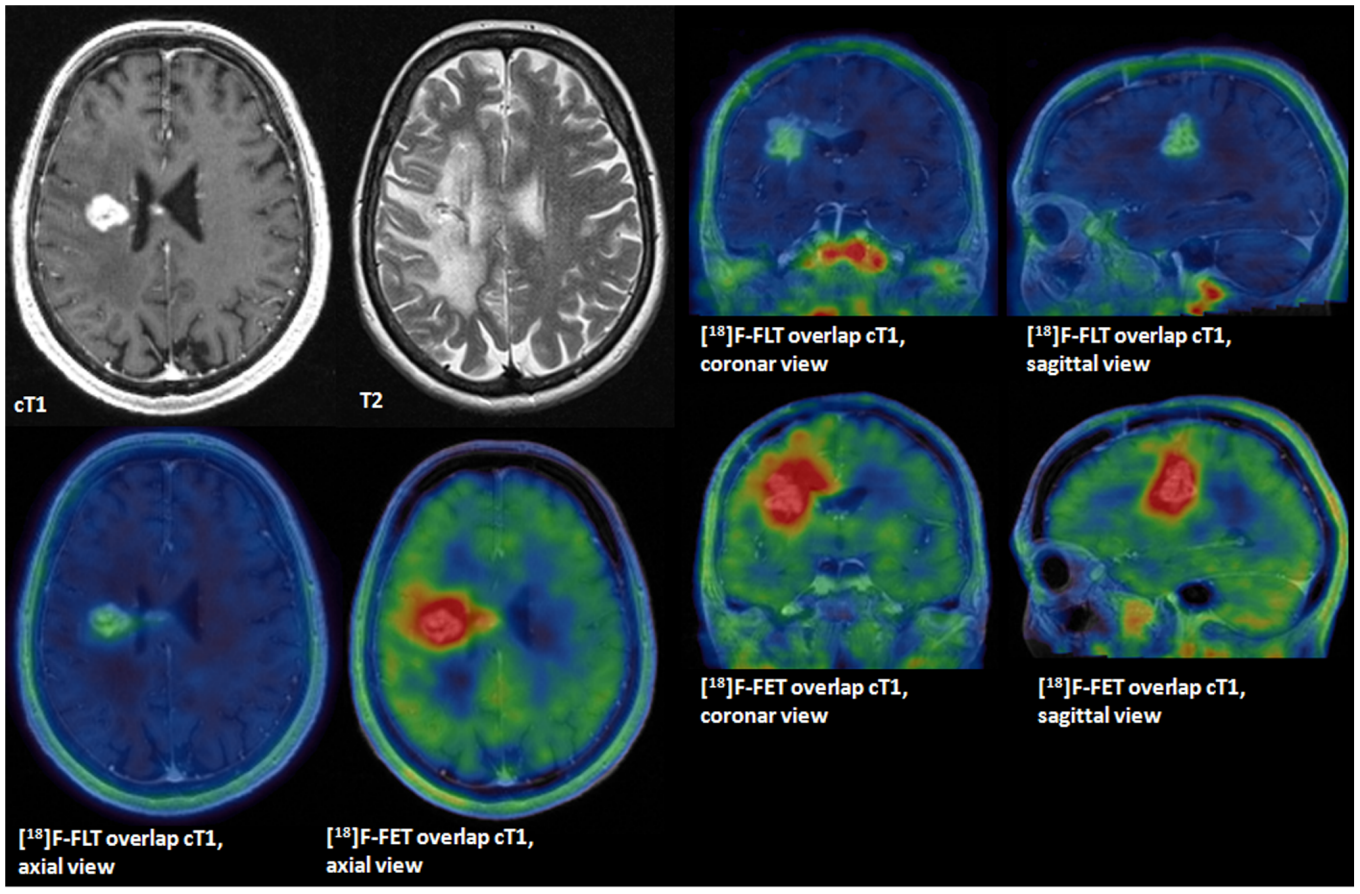
[^18^F]-FET tracer uptake independent from contrast enhancement on MRI, overlap. GBM WHO IV at 2^nd^ tumor recurrence. Contrast enhanced T1 sequences (cT1) tumor volume 5.4 cm^3^, metabolically active tumor volumes for [^18^F]-FET 17.58 cm^3^ and [^18^F]-FLT of 3.9 cm^3^. Overlap analysis detected a [^18^F]-FET volume of 9.2 cm^3^ beyond the borders of cT1.

In 14 of 23 cases (61%) [^18^F]-FET uptake occurred independently from BBB breakdown and was found in non-contrast enhancing tumor areas (mean [^18^F]-FET volume beyond cT1 tumor areas 8.3±2.7 cm^3^ and mean [^18^F]-FET PET tumor volume of the entire tumor 23.9±4.8 cm^3^). [^18^F]-FET tracer uptake was also visible beyond the borders of T2 (8/23 patients, 35%; mean [^18^F]-FET volume beyond T2 volume 1.3±0.2 cm^3^) and beyond [^18^F]-FLT uptake (18/23 patients, 78%; mean [^18^F]-FET volume beyond [^18^F]-FLT 16.0±3.6 cm^3^). Figure 3 gives an example of [^18^F]-FET uptake independent from [^18^F]-FLT uptake and contrast enhancement on MRI.

Comparison of the spatial distribution of [^18^F]-FLT uptake and contrast enhancement on MRI revealed that in 22 of 23 patients (96%) [^18^F]-FLT uptake was exclusively associated with cT1-positive tumor areas. Only in one GBM patient the [^18^F]-FLT volume was slightly larger than the cT1 volume ([^18^F]-FLT volume beyond cT1 volume 2.7 cm^3^). In no case [^18^F]-FLT tracer uptake was found beyond the borders of T2, in 6 cases [^18^F]-FLT tracer was detected beyond the borders of the [^18^F]-FET uptake (Table 2.).

Finally, comparison of mean [^18^F]-FET and [^18^F]-FLT volumes beyond the borders of contrast enhancement in MRI showed that [^18^F]-FET PET is able to detect cT1-negative tumor parts significantly better than [^18^F]-FLT PET (8.3 vs. 2.7 cm^3^, p<0.001, MWU).

## Discussion

The objective of the present study was to directly compare MRI-based morphologic (cT1, T2) with [^18^F]-FET and [^18^F]-FLT PET-based metabolic tumor volumes in HGG patients on an intra-individual basis.

Our results indicate a strong relationship between [^18^F]-FLT tracer uptake and enhanced BBB permeability. [^18^F]-FLT volumes strongly correlated with cT1 volumes and in 96% of tumors [^18^F]-FLT uptake was detected exclusively within the borders of cT1. Furthermore, [^18^F]-FLT tracer uptake was absent in two HGG patients who had only moderate or no contrast enhancement on MRI.

Similar observations were previously reported in a study comparing [^11^C]-MET and [^18^F]-FLT PET, where patients with non-enhancing anaplastic gliomas on contrast MRI showed significant [^11^C]-MET but no [^18^F]-FLT uptake [32]. In addition, in a mouse model [^18^F]-FLT PET was clearly inferior compared to [^11^C]-MET PET investigating angiogenesis of glioblastomas for detecting early tumor development [33].

In order to explain this limited metabolic trapping of [^18^F]-FLT even into proliferative tumor areas of HGG [34], it is necessary to be aware of its mechanisms regulating transport, accumulation, and retention in tissues [35]. On a molecular level [^18^F]-FLT uptake was shown to be associated with the activity of thymidine kinase-1 (TK1), an intracellular enzyme expressed during DNA synthesis of the cell cycle [36]. In line with this mechanism [^18^F]-FLT uptake correlates with the Ki-67 proliferation rate in patients with newly diagnosed HGG [21]. [^18^F]-FLT, however, has to cross the plasma membrane of cells before it can be trapped by TK1. This cellular uptake is mediated by the bidirectional equilibrative nucleoside transporter 1 (ENT1) [35], [37], a widely distributed plasma membrane nucleoside transporter molecule in the central nervous system [37]. Previous studies investigating the kinetic properties of the [^18^F]-FLT tracer in HGG also showed that [^18^F]-FLT uptake predominantly occurs in tumor regions with disrupted BBB [38], [39]. This was supported by a strong correlation between [^18^F]-FLT LBR and the passive tracer influx rate constant *K1* [39], but not with ENT1 expression or the CD34 vascular density score [35].

In the light of the increasing use of anti-angiogenic treatment strategies, such an association of [^18^F]-FLT uptake in tumor tissue dependent on BBB permeability is clinically relevant. Bevacizumab, a monoclonal antibody against VEGF, may lead to a pseudo-normalization of tumor vessels and a functional restoration of the BBB permeability [40]. Recently, [^18^F]-FLT PET was proposed to assess treatment response to anti-angiogenic treatment in recurrent HGG [41], [42]. On the basis of our results, response assessment of anti-angiogenic treatment in HGG solely based on [^18^F]-FLT has to be interpreted with caution.

In contrast, [^18^F]-FET PET was able to identify HGG even in the absence of contrast enhancement on MRI, showing a better sensitivity than [^18^F]-FLT PET for tumor detection in our patient cohort. Hence, our data confirm previous reports showing that [^18^F]-FET PET is more sensitive than [^18^F]-FLT PET to detect HGG [43]. More importantly, in 61% of the patients [^18^F]-FET tracer uptake was found beyond the borders of contrast enhancing tumor on T1-weighted MRI and in 35% even beyond the borders of T2. Previous studies including patients with primary and recurrent GBM [6], [44] as well as gliomas of various malignancy grades and brain metastases [7] showed that both amino acid tracers [^18^F]-FET and [^11^C]-MET PET delineate tumor tissue outside of MRI cT1 and T2 changes.

A [^18^F]-FET PET signal results from specific tracer uptake into tumor and endothelial cells depending on tumor cell density and microvascular density [45] mediated by LAT amino acid transporters [46]. In addition to this predominant specific uptake, to a lesser part amino acid tracer uptake also results from non-specific uptake due to various pharmacokinetic processes associated with a raised BBB permeability (e.g. VEGF-mediated, reactive astrocytosis [47], radiation induced necrosis [48]), variable tumor blood volume and perfusion (“blood pooling effect” [46]). An association between [^18^F]-FET LBR and contrast enhancement on MRI has also been shown recently [49] by our study group.

Limitations of the current analysis are the relatively small patient cohort, the retrospective study design and the lack of a histologic correlation analysis of the recurrent brain tumors. A lack of histologic correlation studies always raises concern on the presence of possible pseudoprogression. However, image analysis was performed in awareness of this radiographic phenomenon and clinical parameters (median time from radiation until tumor progression was 1.9 years) as well as radiation field analysis was highly suggestive for true tumor progression.

In conclusion, BBB breakdown seems to be a prerequisite for [^18^F]-FLT uptake and even HGG with a high proliferation index may be [^18^F]-FLT negative if they lack contrast enhancement on MRI. In contrast, [^18^F]-FET PET imaging may identify areas of active tumor in HGG more accurately than MRI alone. MRI in combination with [^18^F]-FET PET are therefore accurate complementary imaging modalities for tumor volume assessment. These results are clinically meaningful for improved surgery and radiotherapy planning as well as chemotherapy and anti-angiogenic treatment monitoring.
